# The plastid genome of *Herpetospermum pedunculosum* (Cucurbitaceae), an endangered traditional Tibetan medicinal herbs

**DOI:** 10.1080/23802359.2019.1703603

**Published:** 2020-01-10

**Authors:** Chengwang Wang, Xilong Wang, Tamdrin Tseringand, Yu Song, Rongjie Zhu

**Affiliations:** a School of Life Science, Nanchang University, Jiangxi, China;; b Tibet Plateau Institute of Biology, Xizang, China;; c The Technological Developmnet Exchange Service Center of Linzhi, Xizang, China;; d Center for Integrative Conservation, Xishuangbanna Tropical Botanical Garden, Chinese Academy of Sciences, Yunnan, China;; e Institute of Vegetable Sciences, Tibet Academy of Agricultural and Animal Husbandry Sciences, Xizang, China

**Keywords:** *Herpetospermum pedunculosum*, phylogenetic analysis Cucurbitaceae, chloroplast genome

## Abstract

*Herpetospermum pedunculosum* (Ser.) C. B. Clarke is an important traditional Tibetan medicinal plants in the genus of *Herpetospermum*, Cucurbitaceae. To better determine its phylogenetic location with respect to the other Cucurbitaceae species, the complete plastome of *H. pedunculosum* will be reported, which is the first species with plastid genome sequence in the genus of *Herpetospermum*. Its whole genome is 156,531 bp in length, consisting of a pair of inverted repeats (IRs) of 26,147 bp, one large single-copy (LSC) region of 85,878 bp, and one small single-copy (SSC) region of 18,359 bp. There are 128 genes, including 83 protein-coding genes, 36 transfer RNA (tRNA) genes, and 8 ribosomal RNA (rRNA) genes in the plastome. Phylogenetic analysis based on 13 complete plastomes of Cucurbitaceae species showed sisterhood of *H. pedunculosum* and a clade containing *Trichosanthes kirilowii* and *Hodgsonia macrocarpa*, suggesting the close relationship between tribe Schizopeponeae and tribe Sicyoeae in the family Cucurbitaceae.

*Herpetospermum pedunculosum* (Ser.) C. B. Clarke, an endangered traditional Tibetan medicinal herbs in Cucurbitaceae, is mainly distributed at high altitude in Tibet and Yunnan of SW China, Nepal and NE of India (Yang et al. 2003; Zhang et al. 2008; Xu et al. 2015). Its dried ripe seeds, locally called as ‘Se Ji Mei Duo’ in Tibet, were used for treatment of liver diseases (Ma et al. 2019). As annual scandent herbs, it can survive in extreme environment of the Tibetan Plateau, such as strong ultraviolet (UV) radiation, low temperatures, violent winds, drought and low oxygen concentration (Ma et al. 2015; Kim et al. 2018; Zhao et al. 2019). *Herpetospermum pedunculosum* as a medicinal material in China, previous literature mainly focused on phytochemical and pharmacological studies (Yuan et al. 2006; Yu et al. 2014). Just few genes in the chloroplast genome of *H. pedunculosum* have been applied for analyzing phylogenetic relationship in Cucurbitaceae (Kocyan et al. 2007; Schaefer et al. 2009). Therefore, we sequenced and reported the complete chloroplast genome of *H. pedunculosum*, in order to better understand the relationship with other Cucurbitaceae species.

The specimen of *H. pedunculosum* was collected from Linzhi City, Xizang Autonomous Region, China (94°15′ E, 29°09′ N, 3045 m), total DNA was isolated from silica-dried leaves tissues using a modified cetyltrimethyl ammonium bromide (CTAB) protocol (Doyle and Doyle 1987). The voucher specimen was deposited at Herbarium of Tibet Plateau Institute of Biology (XZ) (Accession No. WXL2019080547). Genomic DNA extraction followed by library construction and sequencing on the Illumina Hiseq 2500 was performed by Annoroad (Beijing, China). The complete sequence was annotated using the two software, Geseq (Tillich et al. 2017) and PGA (Qu et al. 2019), and manually checked and corrected by Sequin.

The complete plastid genome of *H. pedunculosum* (GenBank accession MN711716) is 156,531 bp in length with 37.2% GC contents. Its typical quadripartite structure contains a pair of inverted repeat regions (IRs, 26,147 bp) separated by the large single-copy (LSC, 85,878 bp) and small single-copy (SSC, 18,359 bp) regions. There are 128 genes, including 83 protein-coding genes, 8 rRNA genes, and 36 tRNA genes in the plastid. The GC content in LSC, SSC, and IR regions of the plastid are 35.0%, 31.1%, and 42.9%, respectively.

Furthermore, based on 13 published chloroplast genome sequences, we reconstructed a phylogenetic tree (Figure 1) to confirm the evolutionary relationship between *H. pedunculosum* and other species with published plastomes in Cucurbitaceae, with *Corynocarpus laevigata* (NC_014807) as outgroup. Maximum-likelihood (ML) phylogenetic analyses were performed base on MAFFT v7.407 (Nakamura et al. 2018) and IQ-TREE (Nguyen et al. 2015). The ML phylogenetic tree with 99% to 100% bootstrap values at each node highly supported sisterhood of *H. pedunculosum* and a clade containing *Trichosanthes kirilowii* and *Hodgsonia macrocarpa*, suggesting the close relationship between tribe Schizopeponeae and tribe Sicyoeae in the family Cucurbitaceae.

**Figure 1.**
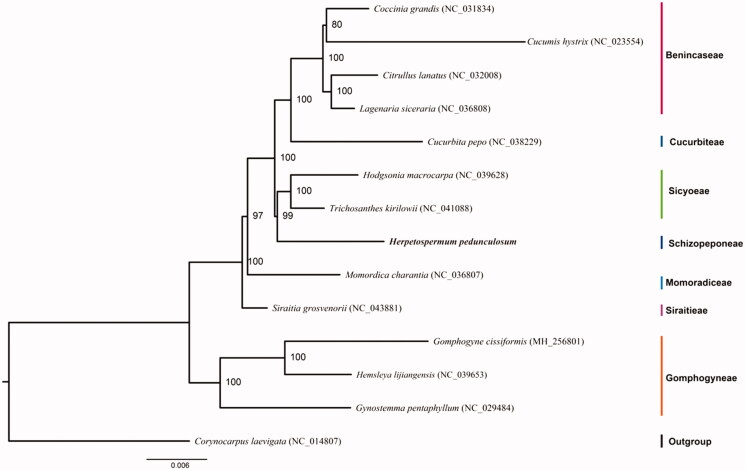
The ML phylogenetic tree showing relationship between *H. pedunculosum* and 13 other species of Cucurbitaceae based on the complete plastid genomes catenated dataset. Numbers in each the node indicated the bootstrap support values.

## Data Availability

The plastome data of the *H. pedunculosum* will be submitted to GenBank (Accession: MN711716).
